# Boundaries of the thoracic paravertebral space: potential risks and benefits of the thoracic paravertebral block from an anatomical perspective

**DOI:** 10.1007/s00276-017-1857-4

**Published:** 2017-04-25

**Authors:** Esther A. C. Bouman, Judith M. Sieben, Andrea J. R. Balthasar, Elbert A. Joosten, Hans-Fritz Gramke, Maarten van Kleef, Arno Lataster

**Affiliations:** 1 Department of Anesthesiology and Pain Management, Maastricht University Medical Center+, P. Debyelaan 25, 6202 AZ Maastricht, The Netherlands; 2 Department of Anatomy and Embryology, Faculty of Health, Medicine and Life Sciences, Maastricht University, Maastricht, The Netherlands; 3 CAPHRI School of Public Health and Primary Care, Maastricht, The Netherlands

**Keywords:** Regional anesthesia, Paravertebral block, Paravertebral space, Clinical anatomy

## Abstract

**Purpose:**

Thoracic paravertebral block (TPVB) may be an alternative to thoracic epidural analgesia. A detailed knowledge of the anatomy of the TPV-space (TPVS), content and adnexa is essential in understanding the clinical consequences of TPVB. The exploration of the posterior TPVS accessibility in this study allows (1) determination of the anatomical boundaries, content and adnexa, (2) description of an ultrasound-guided spread of low and high viscous liquid.

**Methods:**

In two formalin-fixed specimens, stratification of the several layers and the 3D-architecture of the TPVS were dissected, observed and photographed. In a third unembalmed specimen, ultrasound-guided posterolateral injections at several levels of the TPVS were performed with different fluids.

**Results:**

TPVS communicated with all surrounding spaces including the segmental dorsal intercostal compartments (SDICs) and the prevertebral space. TPVS transitions to the SDICs were wide, whereas the SDICs showed narrowed transitions to the lateral intercostal spaces at the costal angle. Internal subdivision of the TPVS in a subendothoracic and an extra-pleural compartment by the endothoracic fascia was not observed. Caudally injected fluids spread posteriorly to the costodiaphragmatic recess, showing segmental intercostal and slight prevertebral spread.

**Conclusions:**

Our detailed anatomical study shows that TPVS is a potential space continuous with the SDICs. The separation of the TPVS in a subendothoracic and an extra-pleural compartment by the endothoracic fascia was not observed. Based on the ultrasound-guided liquid spread we conclude that the use of a more lateral approach might increase the probability of intravascular puncture or catheter position.

## Introduction

The paravertebral block (PVB) is a regional anesthetic technique which revived after the publication of Eason and Wyatt [10] and became very popular especially for thoracotomy [9, 16], breast surgery [4, 28, 29], and inguinal hernia repair [12] during the last 10 years. It may be an alternative to thoracic epidural analgesia as it is as effective as epidural analgesia, has less complications and may reduce the occurrence of postoperative pulmonary complications [9]. Additionally it is more effective than local wound infiltration [3]. However, the most common complications related to its anatomical boundaries are: block failure, intrathecal and epidural spread of local anesthetics, Horner syndrome, intercostal block, pneumothorax and vascular puncture [16, 21].

The thoracic paravertebral space (TPVS) is commonly described as triangular-shaped in transverse cross-section or wedge-shaped in 3D, located bilaterally alongside the whole length of the thoracic vertebral column. The TPVS is filled with fat and is traversed by the dorsal branches and ventral branches of spinal nerves, communicating branches, intercostal nerves and blood vessels, hemi-azygos vein, thoracic duct and sympathetic trunk [10, 11, 14].

The posterolateral aspect of the vertebral column forms the base of the TPVS. The apex of the TPVS communicates with the intercostal spaces laterally. The anterolateral boundary is formed by the parietal pleura and the posterior boundary by the transverse processes of the vertebrae, the head and neck of the ribs together with their interconnecting musculoaponeurotic tissues [8]. The musculoaponeurotic system is formed by the superior costotransverse ligament [30] and the aponeurosis of the internal intercostal muscle (ICM). The psoas muscle at L1 is considered to be the caudal boundary of the TPVS [17]. A cranial boundary is not described.

Although described with these definite anatomical boundaries, the question remains if the TPVS is an as anatomically isolated space as assumed.

The schematic representation of the TPVS as shown in previous publications may be appropriate for general overview and for example teaching purposes, but misses the details shown in an anatomically dissected specimen and lacks precision to serve as a solid base to understand and explain clinical consequences of TPVB. Previous research in cadaver studies using injection of low viscous methylene blue dye (MB) has shown that MB enclosed somatic and sympathetic nerves in the epidural, prevertebral, paravertebral and intercostal spaces [8].

Even dispersion into the abdomen through medial and lateral arcuate ligaments arching over the psoas major muscle was shown [27]. However, due to the use of high volumes of low viscous dye, extensive staining of surrounding structures may impede differentiation. As far as we know the use of a high viscous liquid with a limited spread in this area was investigated only once earlier, [6] using a posteromedial approach with loss-of-resistance (LOR) [10]. In addition, radiological studies with contrast medium [7] and MB [1] showed cranial to caudal spreading of contrast along the paravertebral space, an intercostal dispersion pattern, a combination of both and an intrapleural or cloudy pattern. In a combined radiological and computer tomography study [22] the radiological contrast remained restricted to the TPVS in only 18% of the cases. In other cadaver studies ultrasound was combined with CT scan and radiological contrast to examine the catheter position in the paravertebral space [19, 20]. A correct catheter position was obtained in 11 of 20 cases [19]. With a LOR-technique an adequate catheter position was obtained in 20/26 punctures, whereas an ultrasound-based puncture technique resulted into 24/36 correct positions [20]. Dissection performed in one of the 10 cadavers was not described in detail. Both studies used a posteromedial approach. To obtain needle view during the entire procedure to avoid perforation of the pleura a posterolateral approach was necessary. Aims of this human cadaver study are: (1) optimal determination of the anatomical boundaries, content and adnexa of the TPVS, (2) a description of the ultrasound-guided spread of low and high viscous liquid injection.

To meet the requirements we used a direct comparison between anatomical specimens and schematic illustrations based on these specimens. The observed findings are discussed in relation to clinical relevance.

## Materials and methods

Handwritten and signed codicils from the donors of two formalin fixed thorax specimens and one unembalmed fresh-frozen thawed trunk, as required by the Dutch law, are kept at the Department of Anatomy and Embryology.

The formalin fixed thorax specimens were positioned in supine position and were dissected from the inside to the outside. The anterior thoracic wall, inner organs, major blood vessels, esophagus and trachea were removed. The specimens were rinsed in running tap water. In each specimen, the stratification of the several layers from the inside to the outside was dissected, observed and photographed.

The unembalmed cadaver was positioned in prone position. Level of needle insertion was determined and TPVS was localized by ultrasound using a linear array transducer of 12–5 MHz with an IU 22 US-system (Philips). The orientation was standardized as described below. First, the ribs were identified from caudal to cranial and the different levels were marked. Second, the probe was moved in a sagittal plane from lateral to medial along a specific rib. Third, the costotransverse joint was identified. Fourth, the ultrasound probe was moved in a paramedian and oblique direction, in a way that the image showed the costotransverse transition with the rib of one level lower on the distal-caudal-lateral part. Using this procedure, it should be possible to identify the paravertebral space, the pleura and the aponeurosis of the internal ICM.

**Fig. 1:**
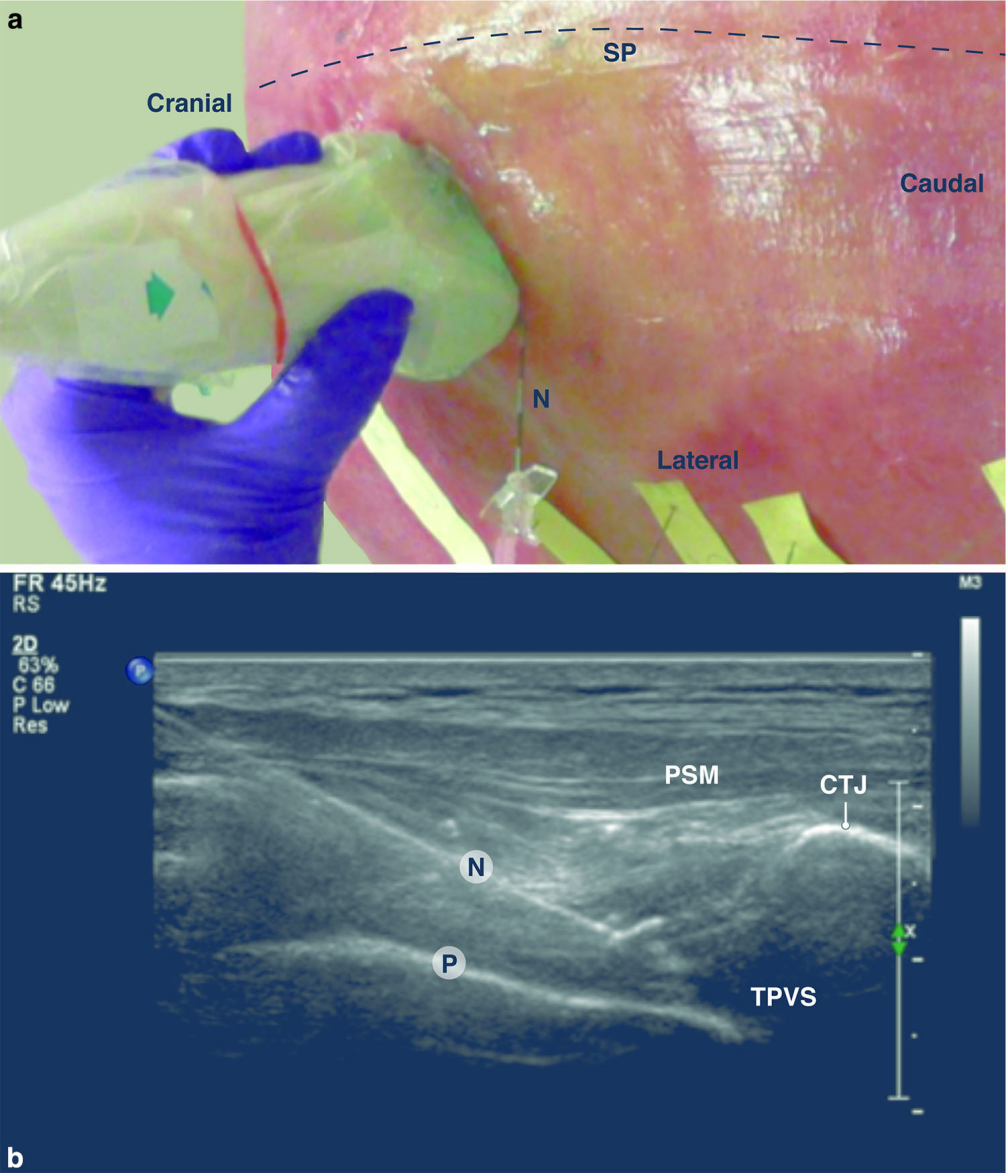
Ultrasound-guided injection. **a** External (posterior) view of unembalmed cadaver specimen showing ultrasound probe position, **b** ultrasound-image (paramedian and oblique view). *CTJ* costotransverse joint, *N* needle, *P* pleura, *PSM* paraspinal muscle, *SP* spinal column, *TPVS* thoracic paravertebral space

Plastic and MB were used to model the dispersion of anesthetic injection fluid in the TPVS. To our knowledge, injection of plastic into the TVPS was only described once earlier [6]; therefore, we decided to compare injection of plastic with that of MB [8]. To overcome extensive plastic and MB dispersion over multiple intercostal levels, three levels of several segments apart at the left side and one level at the right side were selected. The TPVS was localized at the caudal inferior margins of rib 4, 6 and 10 on the left side and of rib 8 at the right side. An 18 g Tuohy needle was inserted from posterolateral in plane to the TPVS, with the bevel oriented to the cranial side (Fig. 1). After confirming the needle position by ultrasound and hydro-dissection (<1, 0 ml of water), 8–10 ml of liquid catalyzed red plastic (Biodur™ epoxyresin E20, Biodur™ hardener E2) was injected on the left side. On the right side an epidural catheter was inserted and through this catheter 5 ml of MB was injected. One physician fixed the ultrasound probe and needle and a second person performed the injection. Dissection of the TPVS was postponed until the next day to allow the plastic to cure and was performed from the posterior and the anterior. Locations of MB, plastic and needles were identified. Because the cadaver had to be turned for the anterior dissection it was necessary to cut the posteriorly protruding Tuohy needles. To consolidate tissues for further dissection and registration of the TPVS structures in detail, it was necessary to fixate the unembalmed trunk by submersion in formalin for several weeks. The process of ultrasound-guided MB and plastic injection and posterior and anterior dissection was registered in photographs.

## Results

The results are presented in two sections: (1) Stratification of the thoracic layers from the inside to the outside by anterior dissection of the formalin fixed thorax specimens, (2) Registration of the dispersion of ultrasound-guided injected dye and plastic by posterior and anterior dissection of the unembalmed trunk, followed by dissection of the TPVS structures in detail in this trunk after submersion fixation with formalin.

### Stratification of the thoracic layers by anterior dissection in the formalin fixed thorax specimens

The following stratification from the inside to the outside was identified and dissected: parietal pleura, endothoracic fascia, innermost ICM, neurovascular bundle (intercostal artery, vein and nerve), internal ICM and external ICM. The innermost ICM was the deep layer of the internal ICM. It was separated from the latter by the neurovascular bundle.

The internal intercostal membrane was in fact the aponeurosis of the innermost ICM. This aponeurosis was continuous with the connective tissue on the inner surface of the rib. The endothoracic fascia was a continuous connective tissue layer between the parietal pleura and the aponeurosis of the innermost ICM.

TPVS boundaries on the anterolateral side were the parietal pleura and the fibroelastic endothoracic fascia, separated from each other by a loose areolar subserous fascia. On the posterior side the TPVS was limited by the transverse process and the superior costotransverse ligament, a thickened medial continuation of the aponeurosis of the internal ICM from the inferior aspect of the transverse process to the superior aspect of the rib tubercle below. Medially the vertebral body, the intervertebral disc and the intervertebral foramen were found as TPVS boundaries. The TPVS appeared to communicate with the contiguous cranial and caudal space, with the epidural space and with the contralateral paravertebral space through the intervertebral foramen or directly through the prevertebral space. Laterally the TPVS was found to be a continuous entity with the intercostal space.

**Fig. 2:**
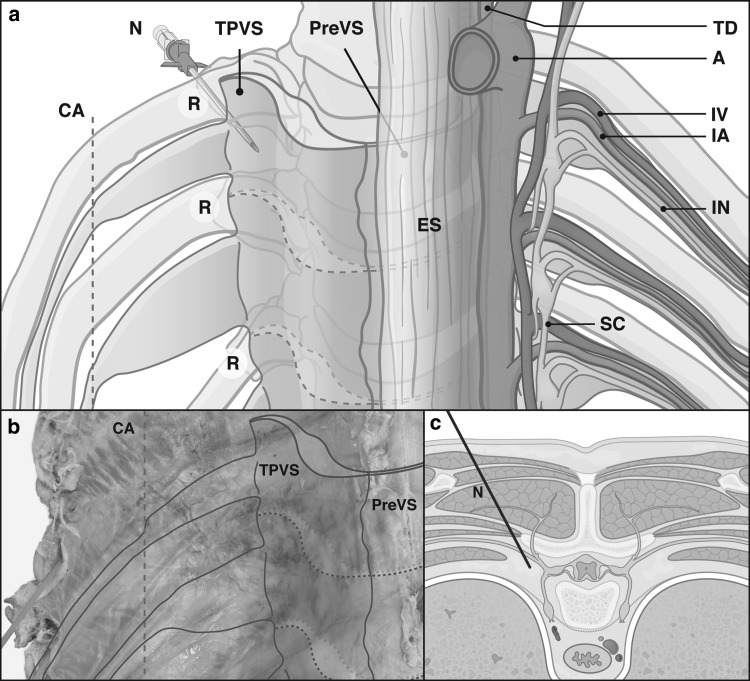
Schematic drawings (**a**, **c**) of thoracic paravertebral space with corresponding photograph of thoracic paravertebral space in formalin fixed thorax specimen (**b**). *A* aorta, *CA* costal angle, *ES* esophagus, *IA* intercostal artery, *IN* intercostal nerve, *IV* intercostal vein, *N* needle, *PreVS* prevertebral space, *R* rib, *SC* sympathetic chain, *TD* thoracic duct, *TPVS* thoracic paravertebral space, **a** and **b** anterior view; **c** transverse view

The TPVS was found to be a non-segmental wedge-shaped fat compartment alongside the thoracic vertebral column, containing the sympathetic chain at a depth of 1 cm. Laterally it extended into lenticular shaped segmentally oriented dorsal intercostal compartments. From the costal angle each dorsal intercostal compartment narrowed to continue as an elongated fat pad along the intercostal neurovascular bundle, inferior to the adjacent rib. Each narrowing fat compartment guided the converging neurovascular bundle to the costal groove (Fig. 2).

### Registration of the dispersion of ultrasound-guided injected MB and plastic by posterior and anterior dissection of the unembalmed trunk, followed by dissection of the TPVS structures in detail in this trunk after submersion fixation with formalin

In the mid-thoracic region, localization of the costotransverse transition, the pleura, the aponeurosis of the internal ICM and the TPVS was easy to achieve at the levels of rib 4 and 6 on the left side and rib 8 at the right side. At the level of rib 10 on the left side the clear identification of the TPVS was difficult because of the different and steep angle of the ribs. The oblique positioning of the probe obviated the disruption of the ultrasound view by acoustic shadowing of the transverse process. However, hydro-dissection improved visibility (Fig. 1).

**Fig. 3:**
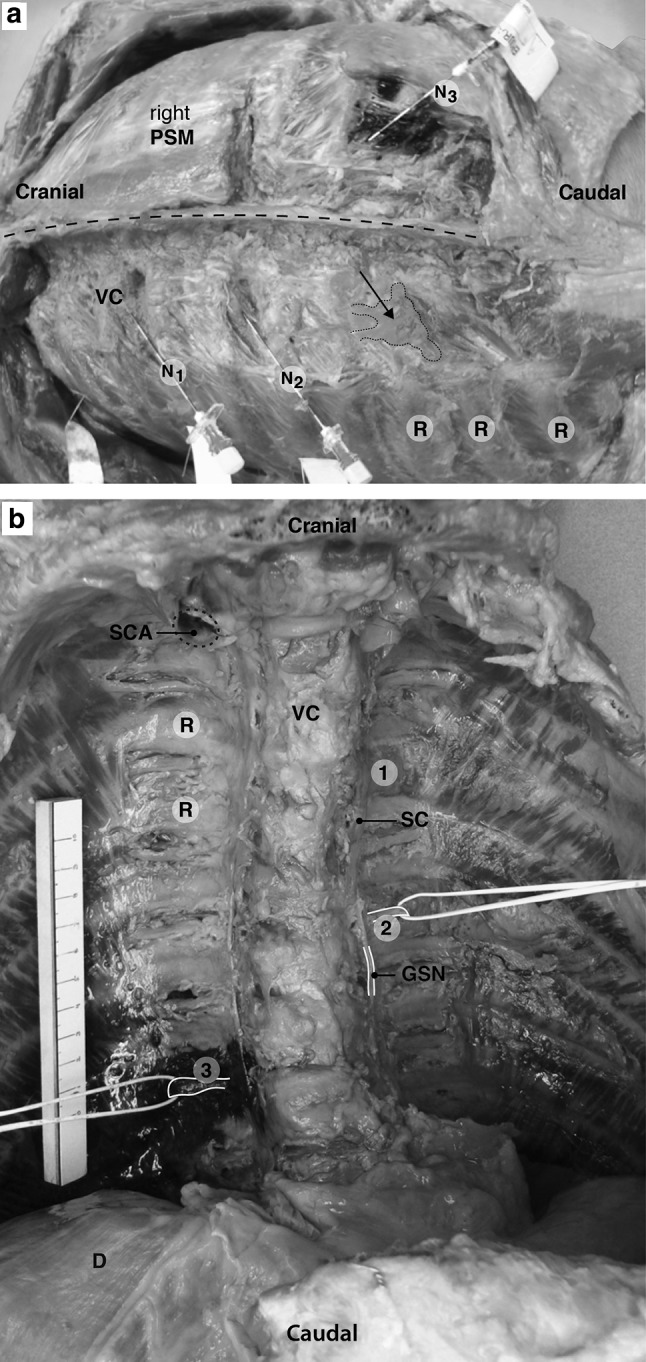
Dissected unembalmed cadaver (**a** posterior view, left paraspinal muscles removed, **b** anterior view, anterior thoracic wall removed). *Arrow* plastic distributed in the erector spinae muscle and external intercostal muscles, *D* diaphragm, *GSN* greater splanchnic nerve, *N* needle, *PSM* paraspinal muscle, *R* rib, *SC* sympathetic chain surrounded by plastic (N1), *SCA* subclavian artery, *VC* vertebral column, *1* intercostal spreading of plastic (N1), *2* intercostal nerve surrounded by plastic (N2), *3* intercostal nerve surrounded by methylene blue (N3)

Dissection from posterior revealed that the ultrasound-guided injection procedure resulted in correct position of the needles at the caudal inferior margins of rib 4 and 6 on the left side and rib 8 at the right side with plastic/MB along the puncture sites (Fig. 3a). MB was visible in the intercostal space on the right side at the level of the insertion site and at one level inferior to the insertion site. At the level of rib 10 on the left side a too superficial position of the Tuohy needle in the external ICM resulted in detachment of the needle and in plastic distributed in erector spinae muscle and external ICM (Fig. 3a).

**Fig. 4:**
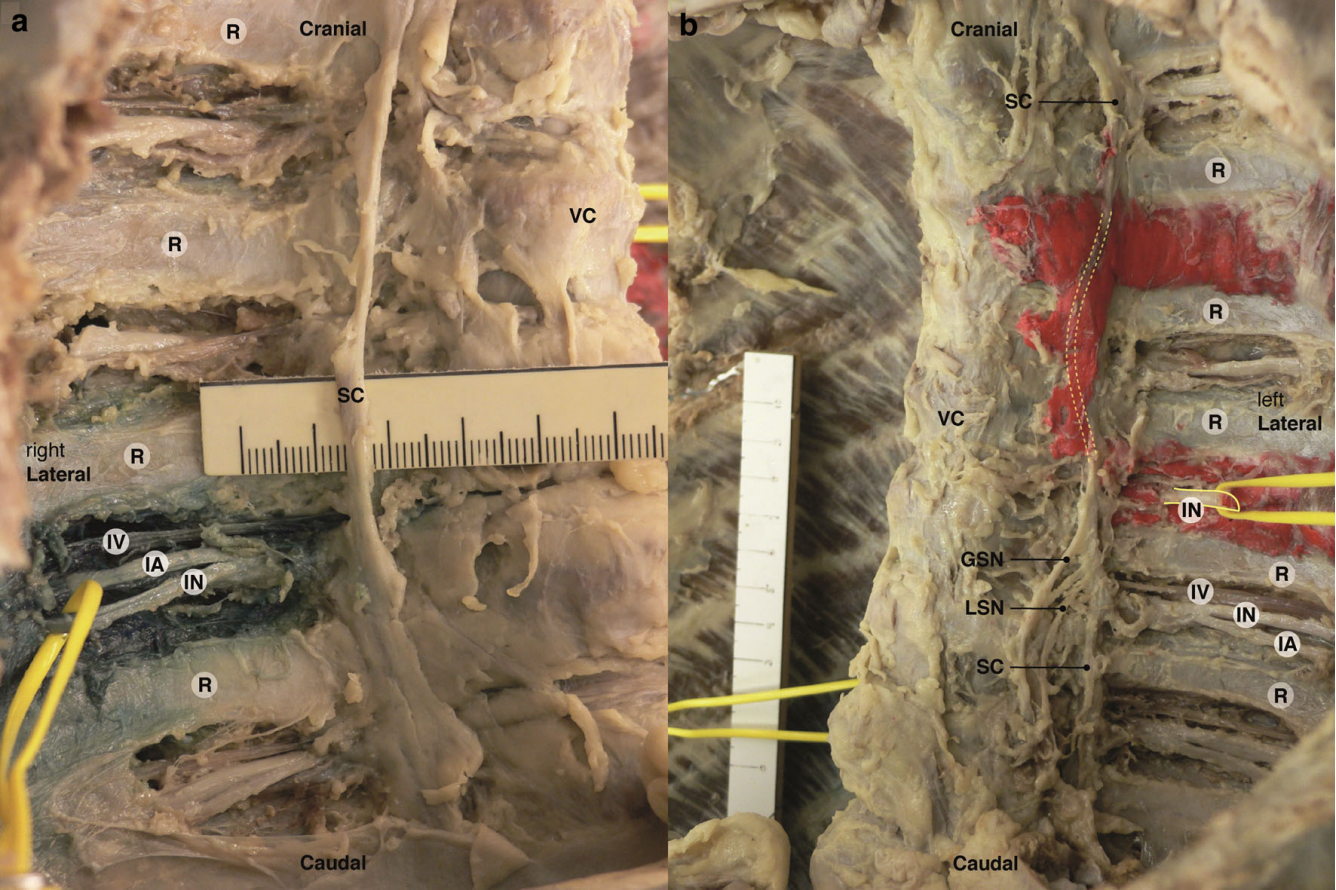
Deep dissection of formalin fixed cadaver (anterolateral view, *right* (**a**) and *left* (**b**) hemi- thorax, anterior thoracic wall removed). *GSN* greater splanchnic nerve, *IA* intercostal artery, *IN* intercostal nerve, *IV* intercostal vein, *LSN* lesser splanchnic nerve, *R* rib, *SC* sympathetic chain, *VC* vertebral column

After the removal of the anterior thoracic wall, inner organs, etc. identification of the needle tips and the injected MB and plastic near anatomical structures was complicated by the presence of blood in the cadaver. The needle tips ended up close to the lung, approximately 0.3 cm. The spread of plastic on the left side and MB on the right side was directed cranially in the TPVS, towards the thoracic prevertebral space, and along the intercostal neurovascular space and stopped at the costal angle. No contralateral dispersion was found, neither with plastic nor with MB. Due to the polymerized, hard plastic used in this technique it was not possible to dissect the epidural space. At the level of rib 10 no plastic was found in the TPVS (Fig. 3b). After rinsing the specimen in running tap water it became clear that the plastic on the left side had spread around the sympathetic chain and laterally beyond the costal angle. The MB on the right side had spread at the injection level surrounding the azygos system, one level caudal along the neurovascular bundle and also posterior to the costodiaphragmatic recess. This was shown more in detail after submersion of the specimen in formalin and subsequent further dissection (Fig. 4a, b).

## Discussion

The aim of this study was to determine the anatomical boundaries of TPVS and to describe the ultrasound-guided observation and spread of fluid-like substances injected from posterolateral in the TPVS. This then will allow understanding of the clinical use and complications of TPVB as performed by posterolateral approach.

We dissected two formalin fixed thorax specimens and an unembalmed thawed trunk. The anatomical boundaries of the TPVS in the two formalin fixed human thorax specimens were found to be relative borders, as the TPVS appeared to communicate with all the surrounding structures. The wedge-shaped space filled with structures is in accordance with previous literature [10, 11, 14]. The TPVS was communicating with the dorsal intercostal compartments, showing a segmental partition. At the costal angle these compartments narrowed, passing into fat pads surrounding the lateral intercostal neurovascular bundles. The separation of the TPVS in a subendothoracic and an extra-pleural compartment by the endothoracic fascia [14, 15] was not observed. The MB had spread around the azygos system and the neurovascular bundle, including the paraspinal venous system. The spread of injected MB dye and plastic in the fresh-frozen cadaver showed that a part of the effect of TPVB can be attributed to the intercostal spread of the local anesthetic. The distribution of the injected dye and plastic makes a hemi-blockade reasonable. The sympathetic chain is commonly included in the area involved. Furthermore, we observed spread posterior to the costodiaphragmatic recess.

Based on literature anatomic variations in TPVS have been known to exist [13]. It can, therefore, not be excluded that our observations are based on such an anatomical variation. In our study, the parietal pleura was located 0.3 cm from the tip of the Tuohy needle. During the opening of the thorax in a cadaver by dissection a pneumothorax occurs, and therefore, it must be assumed that the tip distance to the pleura in the in vivo situation is even smaller. The distance we found from the Tuohy needle to the parietal pleura is in concordance with a clinical study [23], which correlated the ultrasound depth of the parietal pleura with the needle depth necessary to perform an anatomical approach of the paravertebral block. With the technique described it was possible to localize the TPVS and the pleura in the upper thoracic levels in human cadavers. Below the level of the 8th rib the costotransverse joint is situated in a more horizontal position, which hampers the US view.

In line with an anatomical study [27] which showed a spread of dye into the abdomen, we also observed spread of the MB posteriorly into the costodiaphragmatic recess. The communication of the TPVS with the lumbar paravertebral space can be explained by the fact that the abdominal transversalis fascia is in fact a continuation of the endothoracic fascia.

Strength of our study was that we first systematically dissected the TPVS in two formalin fixed thorax specimens and used results of this dissection to copy the clinical execution on an unembalmed human cadaver. The ultrasound approach used was a modified transverse in-plane needle insertion [24] with a linear array transducer of 12–5 MHz. This makes it possible to obtain needle view. The present study differed from an earlier study in human cadavers [8] because to avoid extensive intercostal spread we used a small liquid volume. The combination of two injection substances, MB as an aqueous solution and plastic, as a viscous solution in the same cadaver made it possible to compare the spread of both. This is important because injection of an aqueous solution shows pre-, paravertebral and intercostal spread over multiple segments, whereas isolated injection of viscous solution with limited spread gives a more detailed picture of the local anatomy. The extensive spread of MB along the cranial–caudal axis as described in a previous study of the thoracic vertebral spine [8] was not observed in our study. This might be related to the use of the smaller volume of MB. Furthermore, the injection of MB and plastic was performed with the unembalmed trunk in prone position and as the use of plastic is a different technique and due to the fact that the applied plastic is viscous, we needed a high pressure in the needle to inject the plastic. It is possible that the use of high pressure injection may cause different spreading of solutions. Hence we used two different types of specimen fixation, three separate cadavers and two different injection substrates, which resulted in an optimal and correct analysis of the anatomical boundaries of TPVS.

The use of a cadaver specimen for the clinical execution is also a limitation of this study. LOR-technique or neurostimulation is not feasible in an unembalmed fresh-frozen thawed trunk. Even with an embalmed cadaver using the Thiel method the combination of ultrasound and LOR resulted in only 50% needle tip placement at target [20]. Furthermore, the liquid catalyzed red plastic used is due to the high viscosity not suitable for the LOR-technique. The physical properties of structures identified may have been altered due to post-mortem changes of the TPVS. This makes ultrasound obligatory, although ultrasound in human cadavers differs from ultrasound in living humans, due to this change in physical properties. After all, in general cadaver studies offer the best possible approximation of clinical practice and it is possible to have a very close look at the anatomical spread after dissection.

Our anatomical observations are important to understand clinical consequences of the TPVB. Direct penetration of local anesthetics into the neurological structures as the proposed mechanism of action [26] is supported by the results of our study. The extensive spread of plastic and MB along the sympathetic chain makes the occurrence of a Horner syndrome as a side effect reasonable [5]. The spread along the intercostal nerves may be an anatomical explanation for the abolishment of intercostal somatosensory evoked potentials [25] and the prevention of chronic post-surgical pain [2].

Failure rate and complications with TPVB are not systematically investigated, but some are known from case series [18, 21]. Systematic investigation is hampered by the variety of techniques used. Therefore, we classified the probability of clinical consequences and complications in a 3-point rating scale based on our anatomical findings and on the technique we used (Table 1). The clinical consequences and risk of complications, however, depend on the exact technique of TPVB used: blind performance with LOR, nerve stimulator based, ultrasound-based or surgical techniques will have different implications. The likelihood of pneumothorax is probably higher with a blind or nerve stimulator-based technique than with an ultrasound-based one, whereas inevitable with a surgical technique. Furthermore, the consequences of a complication depend also on the surgery performed. After thoracotomy with thoracic drainage the consequences of a pneumothorax due to a TPVB are minimal, whereas in short stay programs in breast cancer surgery patients it is a major problem. The (hemi)azygos system and intercostal arteries and veins are adjacent to the TPVS. The use of a more lateral approach could increase the probability of intravascular puncture or catheter position, which must be investigated in clinical studies. It is important to extrapolate the findings from this anatomical study to clinical practice and to evaluate this in clinical patient studies, using modern imaging techniques like ultrasound, computed tomography and magnetic resonance imaging. Clinical studies on the feasibility of ultrasound-guided TPVB in patients the effectiveness of it and its side effects are needed. It is of great use to test if the ultrasound technique procedure used to identify the TPVS is suitable for daily clinical practice in terms of efficacy and safety.

**Table 1:** Probability of clinical consequences and complications in a 3-point rating scale based on our anatomical findings and on the technique used

Structures	Spread dye/plastic	Clinical consequences/complications	Clinical likelihood with technique used
Neurovascular bundle intercostal space	Yes	Intercostal block Block failure Vascular puncture	Likely Possible Possible
Endothoracic fascia	Yes	Indistinguishable from parietal pleura	
Parietal pleura	Yes, not penetrated	Pneumothorax	Possible
Intervertebral foramen	Yes	Epidural or intrathecal spread local anesthetics Hematoma	Possible
Prevertebral space	Limited		
Thoracic duct	No	Puncture	Unlikely
Azygos and hemi-azygos vessels	No	Vascular puncture	Possible
Sympathetic chain	Yes	Sympatic blockade horner syndrome	Likely
Paravertebral vessels	Yes	Vascular puncture	Possible

## Conclusions

Based on this anatomical study we conclude that the TPVS is a potential space continuous with the segmental posterior intercostal spaces, the latter being restricted laterally by the costal angle. The separation of the TPVS in a subendothoracic and an extrapleural compartment by the endothoracic fascia was not observed. The ultrasound-guided observation and spread of fluid-like substances injected from posterolateral in the TPVS strongly suggest that a more lateral approach will increase the undesirable probability of intravascular puncture or catheter position.
